# Radiosensitizing the SUMO stress response intensifies single-dose radiotherapy tumor cure

**DOI:** 10.1172/jci.insight.153601

**Published:** 2025-05-22

**Authors:** Jin Cheng, Liyang Zhao, Sahra Bodo, Prashanth K.B. Nagesh, Rajvir Singh, Adam O. Michel, Regina Feldman, Zhigang Zhang, Simon Powell, Zvi Fuks, Richard Kolesnick

**Affiliations:** 1 Laboratory of Signal Transduction,; 2 Laboratory of Comparative Pathology,; 3 Department of Epidemiology and Biostatistics, and; 4 Department of Radiation Oncology, Memorial Sloan-Kettering Cancer Center, New York, New York, USA.; 5 The Champalimaud Center, Lisbon, Portugal.

**Keywords:** Oncology, Vascular biology, Microcirculation, Radiation therapy, Signal transduction

## Abstract

Single-dose radiotherapy (SDRT) is a highly curative modality that may transform radiotherapy practice. Unfortunately, only ~50% of oligometastatic lesions are SDRT treatable due to adjacent radiosensitive normal organs at risk. Here, we address the extent to which an antiangiogenic drug, VEGFR2-antagonist DC101, radiosensitizes SDRT using murine MCA/129 fibrosarcomas and Lewis lung carcinomas, which display a dose range for SDRT lesional eradication virtually identical to that employed clinically (10–30 Gy). SDRT induces unique tumor cure, stimulating rapid endothelial acid sphingomyelinase (ASMase)/ceramide signaling that yields marked vasoconstriction and perfusion defects in tumor xenografts and human oligometastases. Ensuing tumor parenchymal oxidative damage initiates a SUMO stress response (SSR), which inactivates multiple homologous recombination repair enzymes, radiosensitizing all tumor types. While VEGF inhibits neo-angiogenic ASMase, optimal radiosensitization occurs only upon antiangiogenic drug delivery at ~1 hour preceding SDRT. Obeying these principles, we find DC101 radiosensitizes SSR, DNA double-strand break unrepair, and tumor cure by 4–8 Gy at all clinically relevant doses. Critically, DC101 fails to sensitize small intestinal endothelial injury or lethality from the gastrointestinal–acute radiation syndrome. Whereas normal tissues appear not to be under VEGF regulation nor sensitized by our approach, its application might render many currently intractable oligometastatic lesions susceptible to SDRT eradication.

## Abstract 2

Sensitizing the SUMO Stress Response markedly increases single dose radiotherapy-induced double strand break unrepair and tumor cure at all clinically-relevant doses in pre-clinical models

## Introduction

Single-dose radiotherapy (SDRT) is an approach to radiotherapy driven by the technologic advances of online image guidance radiotherapy (IGRT) and intensity-modulated radiotherapy (IMRT), allowing safe delivery of focused ultrahigh ionizing radiation doses to patient tumors. Recent clinical trials demonstrate that SDRT provides a therapeutic advantage compared with conventional hypofractionated stereotactic body radiation therapy (SBRT). A nonrandomized Phase II clinical trial of oligometastatic human cancer showed that 24 Gy SDRT ablates ≥ 92% of metastatic lesions of all types measured at 5 years, whereas ablation was only 38% with a standard of care 3 doses of 9 Gy (3 × 9 Gy) hypofractionation radiotherapy schedule (1). A follow-up prospective, randomized Phase III trial revealed a 3-year cumulative incidence of local relapse after 24 Gy SDRT of 5.8% compared with 22% following 3 × 9 Gy SBRT (*P* = 0.0048) (2)**.**

Mechanistically, we have provided evidence that SDRT engages a different biology than classical fractionated radiotherapy to yield such high local cure rates (3). Whereas the basic lethal lesion induced by ionizing radiation is the DNA double-strand break (DSB) (4, 5), tumor cell death in response to conventional fractionated radiotherapy depends on DSB misrepair, accrued to a larger extent in tumor cells versus surrounding normal tissues during daily fractionated dose regimens as a consequence of deleterious mutations existing within enzymes of the tumor cell DNA repair apparatus (4, 5). In contrast, we showed that SDRT-mediated tumor cure results from concomitant damage to tumor microvasculature and parenchymal tumor cell DNA (3, 6, 7). Specifically, we reported that, beginning at a threshold of about 10 Gy, SDRT engages acid sphingomyelinase (ASMase)/ceramide signaling in tumor microvasculature (3), resulting in an imbalance between the dilatory effects of nitric oxide and the constrictive effects of endothelin (8–10). Massive vasoconstriction-induced perfusion defects ensue that profoundly affect irradiated tumor cells by generating acute hypoxia therein that initiates a cytoprotective SUMO stress response (SSR) (3, 11, 12), previously reported in stroke models (13). This SSR consumes SUMO3, required for SUMOylation and activation of multiple homologous recombination repair (HRR) enzymes (i.e., BRCA1, RAD51) (14, 15), thereby globally inactivating HRR, resulting in unrepair of DNA DSBs (3). Hence, while conventional fractionation tumor cell death depends on progressive build-up of DSB misrepair during cell division, which eventually yields lethal chromosomal dysfunction (4, 5), SDRT induces, at clinically effective dose levels, a cascade of events that leads acutely to unrepair of DSBs that progress into chromosomal aberrations incompatible with cell survival, accounting for the high clinical SDRT cure rate (3).

While SDRT is highly effective, studies of human oligometastatic cancer revealed that only about 50% of oligometastatic lesions are amenable to tumor ablation with the clinically utilized dose of 24 Gy due to interference with treatment delivery by adjacent radiosensitive normal organs at risk (OARs) (16). In such settings, reduction of tumor dose within the collateral tumor subvolume interacting with the OAR would need to be employed to a level below the threshold dose for toxicity specific to that OAR (16, 17). Such tumor dose “sculpting” with fractionated radiotherapy turns out to be incompatible with tumor cure (16, 17) because misrepair-dependent tumor cure is predicated on each cell receiving a high enough radiation dose to accumulate a tumoricidal load of misrepaired lesions (18, 19). Delivery of less than a tumoricidal dose to every tumor cell with dose-sculpting fractionated regimens, thus, results in high risk of local recurrence within the sculpted tumor subvolume (16, 17, 20, 21). In contrast, our preclinical SDRT studies indicate that O_2_ diffuses freely across the interstitial space within the encapsulated tumor volume (3); hence, the marked ASMase/ceramide-dependent acute hypoxia generated within the major portion of the tumor that receives full-dose SDRT equilibrates with the lesser pO_2_ reduction generated in the sculpted subdomain. Such equilibration mitigates the attenuation of the SSR response that would otherwise occur in subdomain tumor cells, permitting sufficient HRR inactivation throughout for tumor cure. Consistent with this notion, our recent Phase II study showed that, if ≥ 60% of tumor receives 24 Gy, dose sculpting to 18 Gy in the remaining ≤ 40% renders 90% local cure at 5 years, while the OAR shows no high-grade toxicity (16). This strategy permitted accrual of an additional 25% of all patients presenting with oligometastases for treatment with curative SDRT (16) — patients who otherwise would require potentially complex surgery, if at all feasible.

Whereas this approach, termed perfusion-modulated dose sculpting (PMDS) (16), permits cure with near certainty in ~75% of oligometastatic lesions, the challenge implicit in this model is to devise a strategy to safely treat the remaining 25% of lesions that abut OARs with a toxicity threshold of < 18 Gy, effectively requiring sensitizing SDRT to below 14 Gy (16). If it were possible to left shift the SDRT curative dose-response curve such that the dose to the OAR would be < 14 Gy, it might be possible to cure nearly all visible lesions using SDRT+PMDS. Since evidence indicates that SDRT-PMDS appears safe, it might be possible to replace surgery or chemotherapy in some clinical settings as first-line therapy.

The current study addresses the extent to which it might be anticipated that an antiangiogenic drug may be capable of radiosensitizing tumor allografts in mice that display a dose-response range similar to that used in clinical SDRT. Our prior studies defined basic parameters for antiangiogenic radiosensitization of ASMase-ceramide mediated SDRT tumor response as follows: (a) VEGF is the principle inhibitor of ASMase in tumors but not in normal tissues (3, 22); (b) antiangiogenic drugs must be given at ~1 hour before SDRT to radiosensitize, since ASMase can only be derepressed for 1 hour (22, 23); and (c) irrespective of class or t_1/2_, all antiangiogenic drugs (anti-VEGF antibodies, VEGFR tyrosine kinase inhibitors, or antagonistic anti-VEGFR antibodies) appear equally effective in ASMase/ceramide-mediated radiosensitization of SDRT (22–24). Based on these principles, the current studies formally address the extent to which it might be possible to radiosensitize SDRT in preclinical models and show that, across all clinically relevant doses, it is possible to achieve 5–8 Gy radiosensitization of SSR-mediated HRR inactivation and tumor cure.

## Results

### Timed delivery of the VEGFR2 inhibitor DC101 yields large radiosensitization of the SDRT tumor response across the clinically relevant dose range.

**Figure 1:**
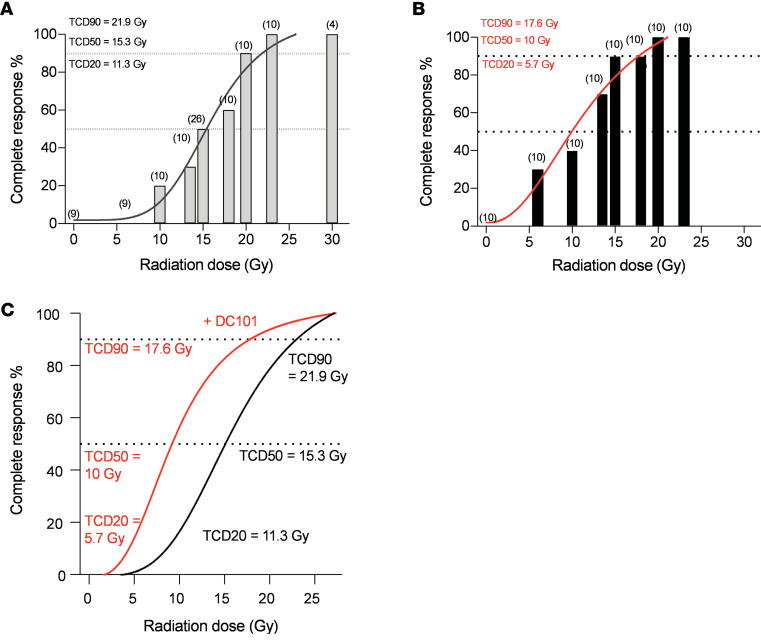
Delivery of the VEGFR2 inhibitor DC101 at 1 hour preceding SDRT is highly radiosensitizing. (**A** and **B**) Mice harboring 100–150 mm^3^ MCA/129 flank fibrosarcomas were pretreated without (**A**) or with (**B**) i.v. DC101 (1,600 µg/25 gm mouse) at 1 hour preceding the indicated SDRT doses. (**C**) Combined cohorts. Tumors undetectable at 120 days were considered complete responses. Parentheses in **A** and **B** indicate number of mice/group. Of note, 15 Gy control data in **A** were previously published in ref. 50. GraphPad Prism (version 8) was used for nonlinear regression analysis as denoted by lines (sigmoidal curve fit) and quantitation of 20%, 50%, and 90% tumor control doses (TCD). R was used for multivariate logistic regression analysis to calculate the OR for each 1 Gy increase of dosage on complete response (OR = 1.44) and the OR for 8 Gy + DC101 versus 8 Gy only on complete response (OR = 6.12). Both effects are statistically significant (*P* < 0.001).

Mice harboring 100–150 mm^3^ murine MCA/129 fibrosarcomas in the flank were subjected to SDRT in the range of 0–30 Gy, which encompasses the entire range of SDRT doses used in the clinic (25). This tumor model was selected after an exhaustive search directed at establishing a preclinical model with an SDRT dose range resembling that observed in humans. One cohort of mice received the VEGFR2 inhibitor DC101 at 1 hour preceding SDRT, a time optimized by our group for derepressing ASMase to sensitize B16F1 melanoma and MCA/129 fibrosarcoma xenografts to a single high-dose radiation exposure (22, 23). DC101 is the murine version of the clinically approved VEGFR2 inhibitor ramucirumab (26). Figure 1, A and B, depict dose-dependent complete responses to SDRT in cohorts without and with DC101 pretreatment, respectively. Histologic analysis at autopsy of the original site of tumor residence before SDRT by a board-certified veterinary pathologist revealed complete responses at 120 days after SDRT represent tumor cures (Supplemental Table 1; supplemental material available online with this article; https://doi.org/10.1172/jci.insight.153601DS1). A multivariate logistic regression based on the total 178 mice was performed to investigate the effect of dose and group. While dose has an odds ratio (OR) of 1.44 for each 1 Gy increase of dosage, the OR for comparing the DC101 group to the radiation alone group is 6.12, meaning that DC101 induces a response with higher probability compared with radiation alone. Both effects are statistically significant (*P* < 0.001). Figure 1C displays combined cohort data that indicate that antiangiogenic drug delivery under conditions optimized for ASMase/ceramide signaling of microvascular dysfunction yields large radiosensitization (4–6 Gy) of SDRT tumor cure at all clinically relevant dose levels. Similar 6–8 Gy radiosensitization was observed in Lewis lung carcinoma (LLC) xenografts with DC101 delivery timed to 1 hour preceding irradiation (Supplemental Figure 1, A and B).

### DC101 radiosensitizes the SSR.

**Figure 2:**
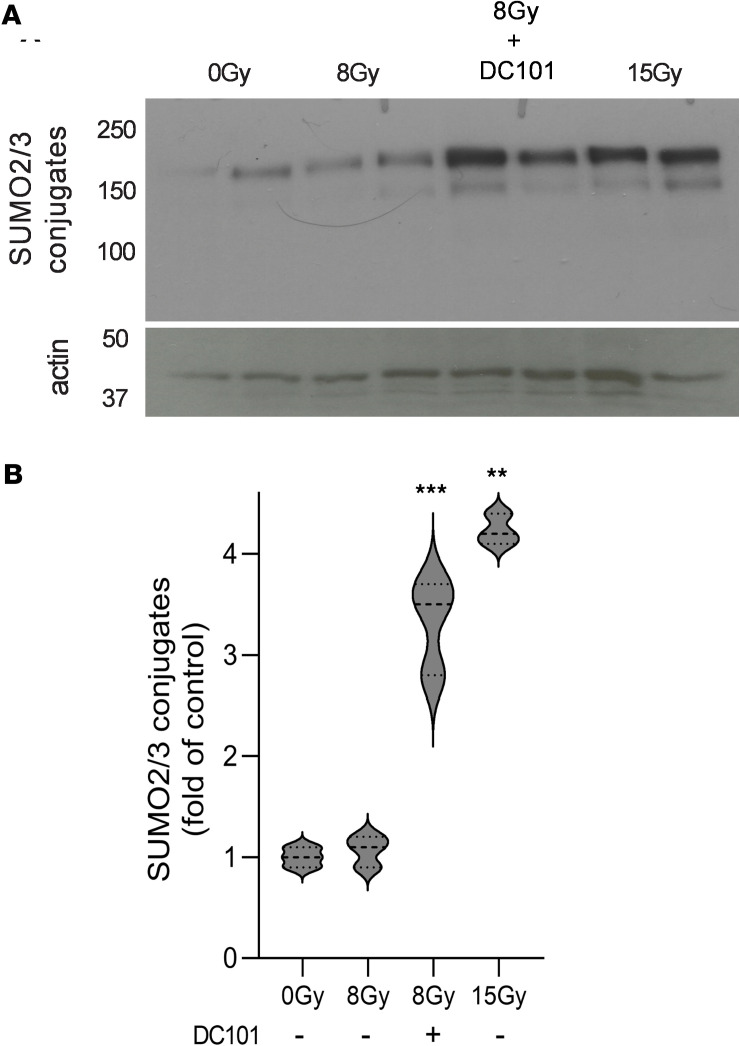
Delivery of the VEGFR2 inhibitor DC101 at 1 hour preceding SDRT sensitizes the SUMO stress response. (**A**) Representative Western blot image depicting high molecular weight (>100 kDa) SUMO2/3 conjugates (upper panel) contained in extracts of MCA/129 flank fibrosarcomas isolated at 3 hours after SDRT from tumors treated as in Figure 1 with or without a 1-hour DC101 pretreatment. Actin serves as a loading control. (**B**) Quantitative analysis of high–molecular weight (>100 kDa) SUMO2/3 conjugates. Data, quantified by densitometry relative to actin loading controls, represent mean ± SD collated from 3 independent experiments using 2 mice/group each. **P = 0.014, ***P < 0.001 versus unirradiated controls (2-sided Wilcoxon rank sum test).

Prior work indicates that SDRT-induced ASMase/ceramide-mediated vascular dysfunction yields large, transient perfusion reduction within minutes in numerous tumors implanted in mice and in a Phase II clinical trial performed at MSKCC in spinal/soft tissue oligometastases (3). In mice, detailed analysis indicates that this leads to large increases in reactive oxygen species in tumor parenchymal cells, resulting in induction of the proteoprotective SSR. Hence, we assessed whether antiangiogenic radiosensitization of MCA/129 fibrosarcoma tumor control conferred concomitant SSR radiosensitization. For these studies, we selected the LD_10_ dose of 8 Gy as shown in Figure 1. The SSR is typically evaluated by formation of high molecular weight (>100 kDa) SUMO-protein complexes detected by Western blot using an antibody that detects SUMO2/3. Figure 2 shows that 8 Gy induces minimal formation of high–molecular weight SUMO complexes at 3 hours after irradiation, whereas 15 Gy, the LD_50_ dose, induces a robust 4.2- ± 0.2-fold increased SUMO2/3 complex formation (*P* = 0.014 versus unirradiated controls). Consistent with the concept that the SSR mediates dysregulation of DNA repair to confer SDRT tumor cure, DC101 enhanced protein SUMOylation by 8 Gy, 3.3- ± 0.5-fold (*P* < 0.001 versus unirradiated controls). Our data demonstrate that the effect of DC101 to enhance SUMOylation also applies at higher radiation doses. Supplemental Figure 2 shows that, compared with 15 Gy alone, DC101 increases protein SUMOylation by 1.4-fold (*P* = 0.025 15 Gy+DC101 versus 15 Gy). DC101 delivered at 1 hour before irradiation similarly enhances SSR in LLC xenografts, with 13 Gy inducing minimal protein SUMOylation, consistent with minimal tumor response, increasing 3.4-fold to a level nearly identical to that induced by the TCD_50_ dose of 25 Gy (Supplemental Figure 1C). These studies provide strong support for the notion that delivery of antiangiogenic drugs timed to enhance ASMase/ceramide-mediated vascular dysfunction leads to a large increase in oxidative damage to tumor cells after SDRT early during the sequence of events leading to increased tumor cure.

### DC101 radiosensitizes misrepair/unrepair of DSBs.

**Figure 3:**
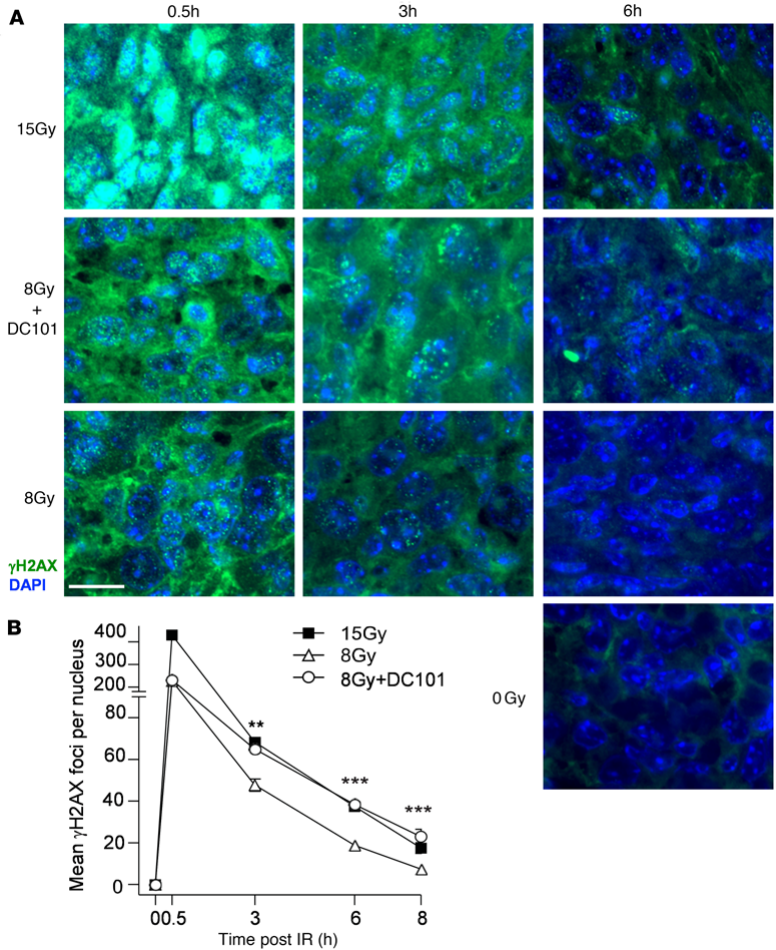
Delivery of the VEGFR2 inhibitor DC101 at 1 hour preceding SDRT attenuates γH2AX focus resolution. (**A**) Representative γH2AX foci in MCA/129 flank fibrosarcomas treated with SDRT as in Figure 1 with or without DC101 pretreatment. Scale bar: 20 µm. (**B**) Data represent mean ± 95% CI, evaluating 3–5 high-powered fields (magnification, ×40) in 2 mice/group. **P = 0.003, ***P < 0.001 8 Gy + DC101 versus 8 Gy (2-sided Wilcoxon rank sum test).

Whereas radioresistant tumors repair DSBs with high fidelity, radiosensitive tissues repair DSBs poorly, resulting in lethal chromosomal aberrations during cell division. Here, we initially quantified resolution kinetics of γH2AX repair foci, the biomarker of choice for assessing global DSB repair in vivo (27). Foci colocalizing γH2AX with MDC1 and/or 53BP1 are considered specific for DSB repair, formed within minutes of DSB induction and resolving once DSB repair is accomplished. Figure 3 shows that, prior to irradiation, γH2AX foci are undetectable and that, at 30 minutes after 15 Gy SDRT, MCA/129 fibrosarcomas display approximately 400 foci per cell, whereas 8 Gy–treated tumors display about half that amount with or without DC101. Whereas during resolution of γH2AX foci, the 15 Gy–treated specimens show a doubling of foci compared with the 8 Gy specimens at all time points, DC101 pretreatment to engage ASMase-ceramide vascular dysfunction renders 8 Gy repair indistinguishable from 15 Gy at all times from 3 to 8 hours. DC101 delivery at 1 hour before 13 Gy treatment of LLC xenografts similarly slows γH2AX focus resolution to a pattern nearly identical to that observed with the TCD_50_ dose of 25 Gy (Supplemental Figure 1D). Furthermore, DC101 also radiosensitizes higher radiation doses, delaying γH2AX focus resolution in MCA/129 fibrosarcomas after 15 Gy to levels statistically indistinguishable from 20 Gy (Supplemental Figure 3, A and B) at 3 hours after irradiation.

**Figure 4:**
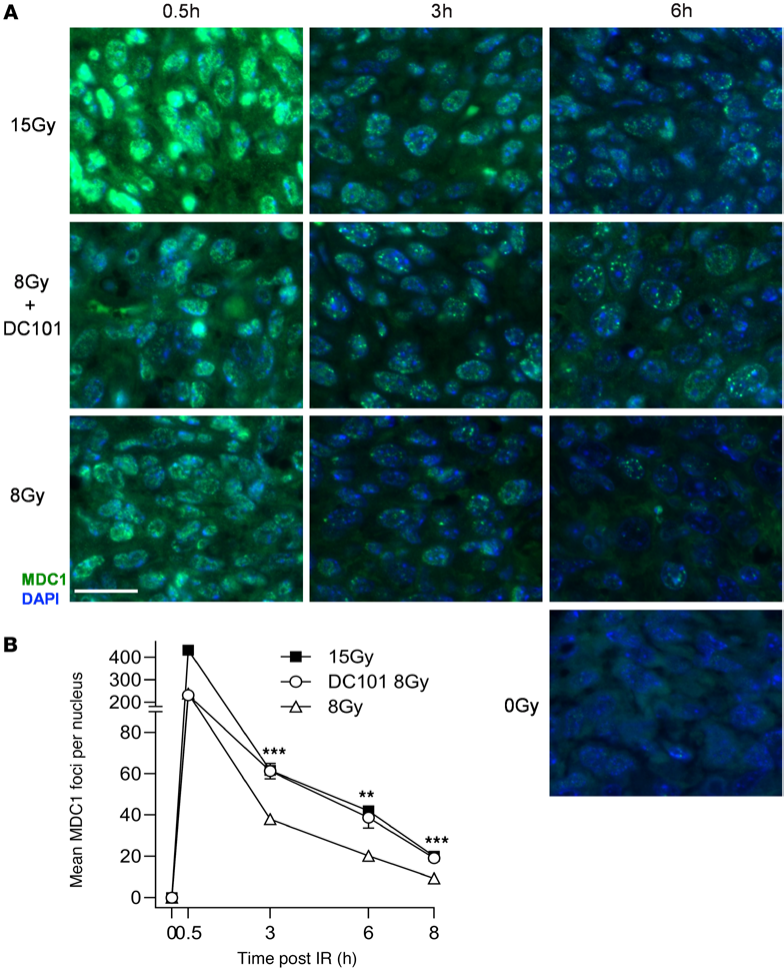
Delivery of the VEGFR2 inhibitor DC101 at 1 hour preceding SDRT attenuates MDC1 focus resolution. (**A**) Representative MDC1 foci in MCA/129 flank fibrosarcomas treated with SDRT as in Figure 1 with or without DC101 pretreatment. Scale bar: 20 µm. (**B**) Data represent mean ± 95% CI, evaluating 3–5 high-powered fields (magnification, ×40) in 2 mice/group. **P = 0.002, ***P < 0.001 8 Gy + DC101 versus 8 Gy (2-sided Wilcoxon rank sum test).

Figure 4 shows comparable data examining the accrual and resolution of MDC1 foci. Prior studies from our program indicate that γH2AX and MDC1 colocalize in the same foci by confocal microscopic analysis (3). As with γH2AX, no MDC1 foci are detected before irradiation. At 0.5 hours after 15 Gy, MCA/129 fibrosarcoma specimens accrue about twice as many MDC1 foci as 8 Gy foci, a doubling that is maintained during the 8-hour repair period. Most importantly, DC101 pretreatment converts the 8 Gy pattern of repair to one indistinguishable from 15 Gy. As with γH2AX, DC101 also radiosensitizes higher radiation doses, delaying MDC1 focus resolution after 15 Gy to levels statistically indistinguishable from 20 Gy (Supplemental Figure 3, A and C) at 3 hours after irradiation.

**Figure 5:**
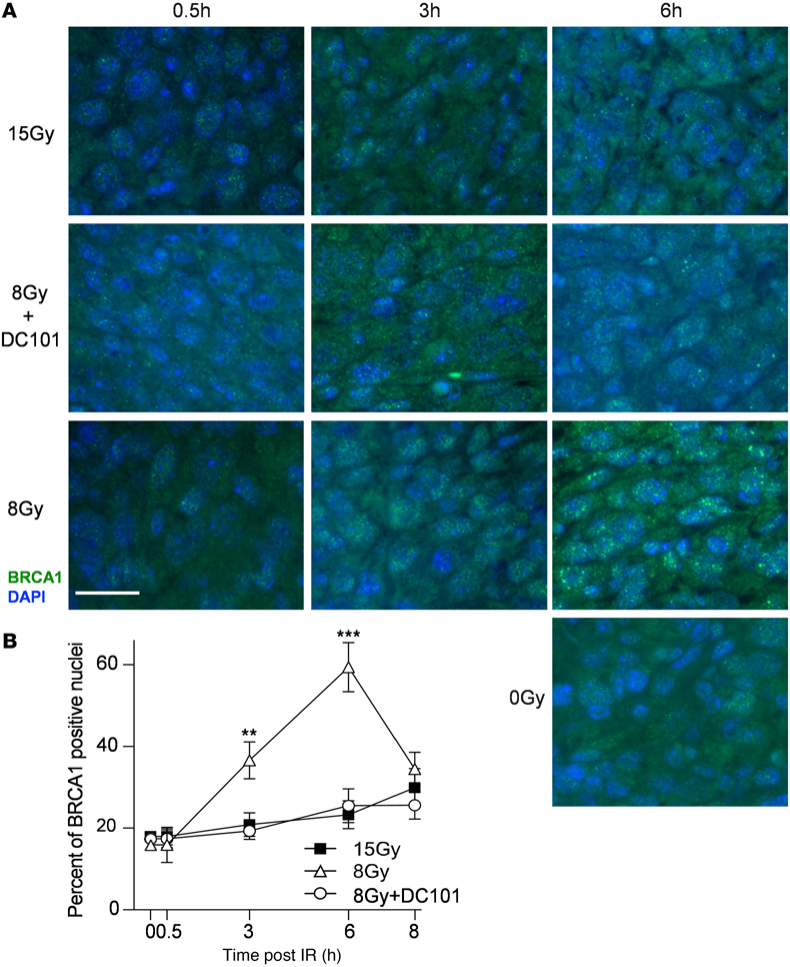
Delivery of the VEGFR2 inhibitor DC101 at 1 hour preceding SDRT prevents loading of BRCA1 into DNA repair foci. (**A**) Representative BRCA1 foci in MCA/129 flank fibrosarcomas treated with SDRT as in Figure 1 with or without DC101 pretreatment. Scale bar: 20 µm. (**B**) Data represent mean ± 95% CI, evaluating 3–5 high-powered fields (magnification, ×40) in 2 mice/group. **P = 0.003, ***P = 0.001 8 Gy versus 8 Gy + DC101 (2-sided Wilcoxon rank sum test).

**Figure 6:**
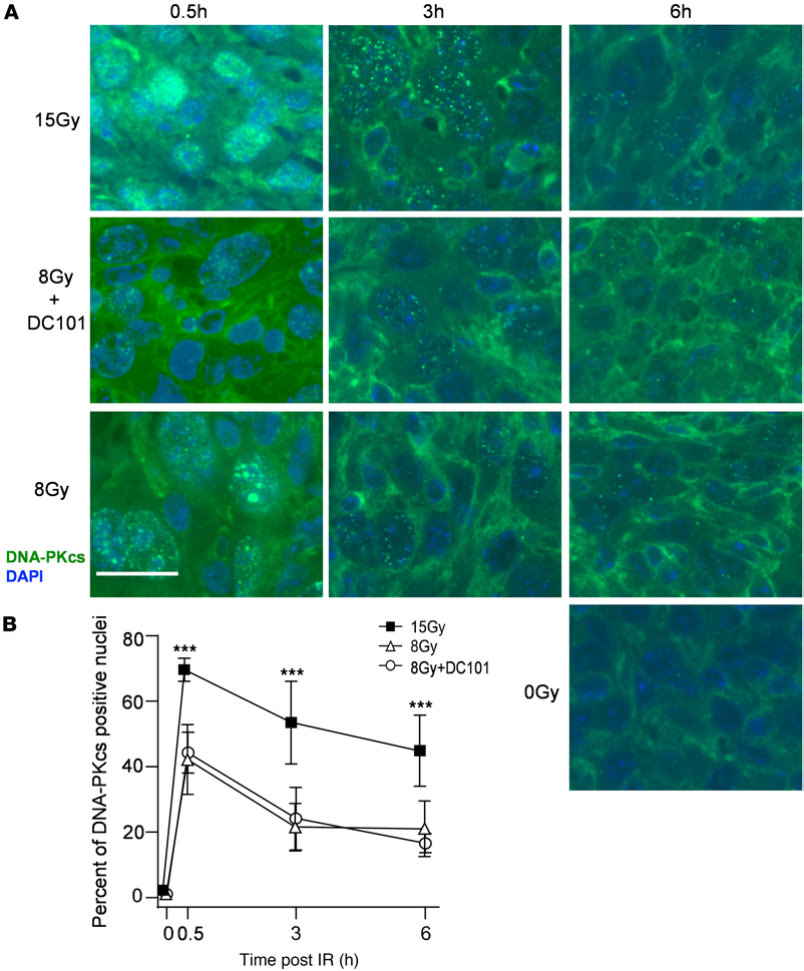
Delivery of the VEGFR2 inhibitor DC101 at 1 hour preceding SDRT does not affect accrual or resolution of DNA-PKcs repair foci. (**A**) Representative DNA-PKcs foci in MCA/129 flank fibrosarcomas treated with SDRT as in Figure 1 with or without DC101 pretreatment. Scale bar: 20 µm. (**B**) Data represent mean ± 95% CI, evaluating 3–5 high-powered fields (magnification, ×40) in 3 mice/group. ***P < 0.001 15 Gy versus 8 Gy or 8 Gy + DC101 (2-sided Wilcoxon rank sum test).

To confirm that HRR inactivation is the target of DC101-induced ASMase-mediated DNA damage repair dysfunction, we examined the pattern of BRCA1 focus accrual and resolution in SDRT-irradiated MCA/129 tumors (Figure 5). As we previously published (3), a modest number of BRCA1 foci are detected prior to irradiation that increases slowly after the largely noncurative dose of 8 Gy SDRT (Figure 1), beginning at 3 hours and peaking at 6 hours. In contrast, as previously shown in this tumor model (3), BRCA1 fails to load into DNA repair foci after the TCD_50_ dose of 15 Gy, an event dependent on induction of the ASMase-ceramide–mediated SSR. Consistent with the attenuation of DNA DSB repair observed with γH2AX and MDC1 foci, DC101 converts the 8 Gy pattern into the 15 Gy pattern, abolishing BRCA1 incorporation into repair foci. In contrast, there was no difference in repair focus accrual or resolution of DNA-PKcs, a principal component of NHEJ repair, upon administration of DC101 with 8 Gy, indicating that antiangiogenic radiosensitization of SDRT is likely, independent of NHEJ entirely (Figure 6).

**Figure 7:**
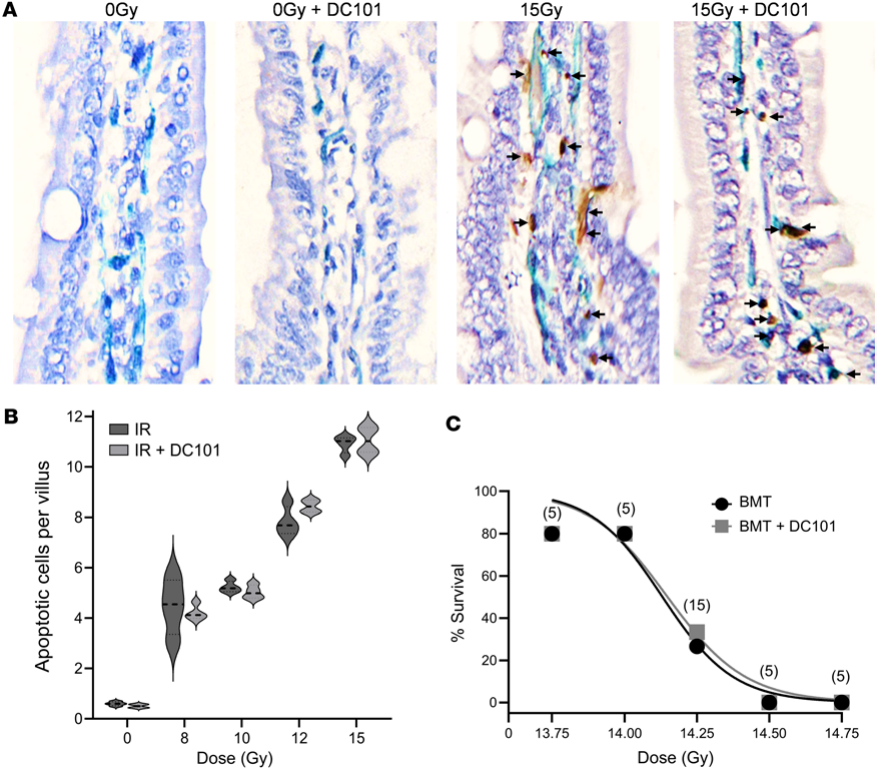
DC101 does not sensitize small intestinal endothelial cell apoptosis or increase animal mortality. C57BL/6J mice, pretreated with 1,600 µg DC101/25 gm mouse via tail vein injection at 1 hour before the indicated radiation doses to derepress ASMase, were sacrificed at 6 hours after radiation. (**A**) Representative bright-field images (magnification, ×100) of individual small intestinal villi double stained with MECA-32 (dark blue plasma membrane signal) and TUNEL (brown nuclear signal) to identify endothelial cells undergoing apoptosis (arrows). Images are derived from 5 μm–thick proximal jejunum sections. (**B**) Quantitation of dose-dependent radiation–induced intestinal endothelial cell apoptosis in mice treated as in **A**. Data (mean ± SEM) are derived from 150–200 imaged fields (magnification, ×40) across 4 mice per group collated from 2 independent experiments. (**C**) Pretreatment with DC101 at 1 hour prior to whole-body irradiation does not sensitize C57BL/6J mice lethality across the entire established gastrointestinal-acute radiation syndrome dose range (29). Of note, all mice received bone marrow transplant (BMT; 5 × 10^6^ cells/25 gm mouse) at 16 hours after irradiation. Numbers of animals per group are in parentheses.

Whereas we originally considered the SDRT vascular affect as mediated via endothelial cell apoptosis (7), in a recent publication, we showed that acute ASMase-ceramide initiated NOX2-dependent microvascular vasoconstriction leading to perfusion deficits and the SSR are the events mediating the SDRT curative effect independent of endothelial cell apoptosis (3). In contrast, we have provided a large body of evidence indicating that apoptosis of normal nonangiogenic endothelial cells mediates the gastrointestinal–acute radiation syndrome (GI-ARS) (28, 29), a lethal outcome of high-dose radiation exposure to the small intestinal microvasculature such as might occur after a nuclear accident or a limited nuclear attack on an American urban center. Consistent with these latter observations, and in contrast to SDRT tumor response, we show here that neither endothelial cell apoptosis (Figure 7, A and B) nor lethality from the GI-ARS (Figure 7C) are affected by DC101 pretreatment. We posit that the potential clinical benefit of antiangiogenic enhancement of SDRT tumor cure will rely on both intensification of tumor-specific SSR vascular biology and lack of engagement of microvascular injury to adjacent normal OARs.

## Discussion

Whereas we recently proposed a mechanism by which endothelial cell ASMase/ceramide activation triggers a cascade of events that link in trans to tumor cell SSR-mediated inactivation of HRR (3), timed delivery of antiangiogenic drugs to optimally derepress VEGF-inhibited ASMase in tumor microvasculature (3, 22–24) should, if our concepts are correct, enhance SSR-induced HRR inactivation and improve tumor response. In fact, our present data show that at least a 5–8 Gy radiosensitization of this SSR biology is possible at the clinically relevant dose range, which would represent a very large clinical effect if translated as is to patients. Therefore, the current study provides additional support for the notion that SDRT uses a biology distinct from that engaged by conventional fractionation to treat cancer, a dual target biology involving a “bystander” microvascular dysfunction that yields a synthetic effect by attenuating tumor cell HR-mediated DNA damage repair, leading to the high clinical success of SDRT.

 The major factor that limits radiotherapy to a secondary role in cancer therapy is proximity of normal tissue. Inability to employ fractionated radiotherapy as first-line therapy in many settings results from proximity of normal OARs that are more radiosensitive to ultrahigh SDRT than adjacent tumor. While baseline SDRT technology minimizes the amount of surrounding normal tissue exposure to the prescribed tumor dose, tumor therapy in multiple clinical settings is restricted by radiosensitivity of the so-called serial normal organs (e.g., CNS structures and major nerve trunks, the pulmonary bronchial tree, GI organs, and the bladder wall), which exhibit organ-specific threshold intolerance within the range of 14–18 Gy (30, 31). The magnitude of the serial OAR toxicity may be substantial (32–35); hence, selective dose reduction in a tumor subvolume adjacent to the OAR may be unavoidable, limiting extended use of this highly curative technology (17, 36). Whereas prevention of serial organ toxicity is prioritized as a basic guideline in radiation treatment planning (17), resolution of serial organ/PMDS incompatibility is nonetheless mandatory for deployment of SDRT as an approach to optimize human tumor cure.

The search for a toxicity-free approach that resolves intractable tumor PMDS/OAR incompatibilities represents a challenge for translational research. At present, the therapeutic mainstay of primary localized human cancer is surgery, while metastatic cancer is a chemotherapy-managed disease. SDRT, however, is gradually emerging as a preferred alternative due to its ability to render, when feasible, an unprecedented 92%–97% toxicity-free eradication of human tumor lesions, regardless of type, size, or tumor target organ (1). In this regard, SDRT compares favorably with systemic chemotherapy because tumors frequently are inherently resistant to a given drug or acquire resistance (37), with consequent uncertainties in treatment outcome. Within this context, PMDS studies are highlighted as providing an unorthodox resolution to frequent limitations to SDRT, expanding catchment of half of lesions previously considered SDRT intractable. Furthermore, SDRT may have an unanticipated benefit, since a recent prospective Phase III clinical trial of 24 Gy SDRT effect on oligometastatic cancer versus standard-of-care 3 × 9 Gy hypofractionated SBRT showed SDRT yielded superior 3-year local control associated with 4-fold reduction in widespread metastatic dissemination (2), suggesting that early ablation of clinically overt oligometastatic lesions with SDRT may reduce polymetastatic spread by an as-of-yet unknown mechanism. SDRT is also cost effective, requiring a single short ambulatory treatment visit, providing patients with a friendly encounter with ablative cancer therapy, as opposed to morbidity-associated tumor-ablative surgery.

The present studies provide compelling evidence that enhanced ASMase activation via timely applied antiangiogenic drug therapy may serve as a radiosensitizing “bio-booster” of PMDS, enabling 5–8 Gy left shift of the SDRT dose-response curve, potentially decreasing the PMDS/OAR interaction from 18 Gy to < 14 Gy and thereby resolving a large fraction of current intractable tumor/OAR settings. Importantly, DC101 does not appear to radiosensitize normoxic normal tissue, as damage to the small intestine at 8–15 Gy — the range of doses that yields morbidity and mortality from the GI-ARS in C57BL/6 mice (6, 28, 29) — is unaffected. An alternative approach to radiosensitization might be via *asmase* gene therapy, employing an adenoviral vector such as *Ad5H2E-mVEGFR2-ASMase* that utilizes a preproendothelin promoter activated exclusively in dividing tumor neoangiogenic endothelium to overexpress ASMase therein to provide a therapeutic ratio (24). While *Ad5H2E-mVEGFR2-ASMase* renders a 6 Gy radiosensitization, deescalating the ablative SDRT dose in MCA/129 fibrosarcoma allografts in mice from 20 Gy to 14 Gy (24), the gene therapy approach appears to be disfavored due to its complexity and higher cost.

Dating back to the original discoveries of Judah Folkman, it has been recognized that hypoxic tumor tissue is under VEGF regulation while normoxic normal tissue is by and large VEGF independent (38–40). Radiation-induced tumor cure is limited in many tumors by excessive VEGF, which results in a friable dysfunctional microvasculature that perfuses tumor parenchyma poorly, yielding hyporesponsiveness to both chemotherapy due to poor drug delivery and radiation due to low ambient oxygen. A substantive preclinical and clinical literature using antiangiogenic agents, including DC101 (41–45), to radiosensitize either single dose or fractionated radiotherapy exists. This literature endorses a strategy designed to continuously repress VEGF in order to normalize this dysfunctional vasculature, improving anticancer treatment, a concept pioneered by Rakesh Jain (42, 46, 47). Unfortunately, these studies have not by and large generically sensitized human cancers to therapy.

Our observation show that scheduling of antiangiogenic drug delivery precisely at 1 hour prior to anticancer therapy to derepress ASMase/ceramide signaling of endothelial cell injury and enhance tumor response differs from this conventional use of antiangiogenic drugs and has yet to be tested clinically. The current studies that show large SDRT-induced radiosensitization of tumor response in the absence of normal tissue response add support for the concept that timed delivery of antiangiogenic drugs to derepress ASMase-ceramide signaling in combination with PMDS might make it possible to cure even those tumors adjacent to OARs with low thresholds for radiation toxicity, a concept we intend to test clinically in the near future.

We regard SDRT-PMDS as a potential advance in human cancer cure that may extend beyond the field of radiation oncology. While SDRT and SDRT-PMDS can currently cure only ≥ 75% of all macroscopic oligometastases, translation of new biological knowledge, as described here, to cure the remaining 25% of lesions with intractable serial OAR constraints may optimize SDRT as a therapeutic lead in multiple types of primary and early metastatic human cancers.

## Methods

### Sex as a biological variable.

Sex was not considered as a biological variable, as findings were expected to generalize across sexes based on prior mechanistic data obtained in similar tumor response models.

### Mice.

sv129/BL/6 male mice, 8–12 weeks old, bred in-house and C57BL/6J mice (The Jackson Laboratory, stock no. 000664) were used in the studies.

### Tumors, irradiation, and tissue preparation.

Murine MCA/129 fibrosarcoma cells (48) and LLC cells (49) were cultured in DMEM high glucose, pyruvate (Thermo Fisher Scientific, 11995073), 10% FBS (BenchMark), and 100 U/mL penicillin-streptomycin (Thermo Fisher Scientific, 15140-122). To produce tumors, 1 × 10^6^ fibrosarcoma cells in 100 mL PBS or 1.5 × 10^6^ LLC cells in 50% Matrigel in 100 mL PBS were injected into the lateral abdominal s.c. tissue as described (50). DC101 (BE0060, BioCell), a rat monoclonal anti–mouse VEGFR2 IgG, was administered at 1,600 µg/25 gm mouse via tail vein injection 1 hour prior to irradiation (51). Tumor volume was monitored using a sliding jaw caliper and quantified a hemiellipsoid approximation V = π/6 abc with a, b, and c denoting the 3 perpendicular diameters (52). Tumors were irradiated at a size of 100–150 mm^3^ as described (50) using a Pantak Siefert Systems X-ray 320 machine (1.17 Gy/min). A cylindrical lead jig was devised to allow exposure of tumor only. Ketamine (0.1 mg/kg) and xylazine (0.02 mg/kg) sedation were employed. For immunofluorescence (IF), tumor specimens were fixed in 4% fresh paraformaldehyde (Electron Microscopy Sciences, 15710) and embedded in paraffin. For Western blotting, tumor specimens were snap frozen in liquid nitrogen and stored at –80°C.

### Quantification of SUMO2/3 in whole tumor lysates.

Whole tumor cell extracts from fresh frozen specimens were generated as described (3, 13). Briefly, frozen tumors were ground with a rotor homogenizer in lysis buffer containing 50 mM β-glycerophosphate (Sigma-Aldrich, G9422), 1 mM EDTA (Thermo Fisher Scientific, 15575020), 1 mM EGTA (Sigma-Aldrich, E4378), 0.5 mM sodium orthovanadate Na_3_VO_4_ (Sigma-Aldrich, 450243), 1% Triton X-100 (Sigma-Aldrich, T8787), and 2% sodium dodecyl sulfate solution (SDS) (Invitrogen, 15553-027) supplemented with Protease Inhibitor Cocktail Complete, Mini, EDTA-free (Sigma-Aldrich, 11836170001), 20 mM N-ethylmaleimide (Sigma-Aldrich, E3876), and 20 mM iodoacetamide (Sigma-Aldrich, I6125). Lysates were immediately boiled at 99°C for 5 minutes and then centrifuged at 14,000*g* for 10 minutes at 4°C. For evaluation of SUMO2/3-conjugated protein complexes, Western blotting analysis was employed. After protein concentration was measured using DC protein assay (Bio-Rad, 500-0114), samples were boiled for 5 minutes in sample buffer without β-mercaptoethanol, subjected (15–50 μg/lane) to SDS-PAGE (6%–15%) separation, and transferred onto immunoblot polyvinylidene fluoride (PVDF) membrane (Bio-Rad, 1620177). Western blot was performed using mouse monoclonal anti-SUMO2/3 (MBL [clone1E7], M114-3, dilution 1:2,000), rabbit polyclonal anti–β-actin (BioLegend, 622102, dilution 1:2,000), and mouse monoclonal anti–α-tubulin (Abcam, ab7291, dilution 1:10,000) antibodies. Anti-mouse HRP (GE Healthcare, NA931, dilution 1:10,000), anti-rabbit HRP (GE Healthcare, NA934, dilution 1:20,000), and anti-mouse HRP (Cell Signaling Technology, 7076S, dilution 1:20,000) were used for secondary antibody detection. The detection signal was developed using Western blotting Detection Reagent (GE Healthcare, RPN2106) and exposed to autoradiography films (Labscientific, XAR ALF 2025). Densitometric analysis was carried out using ImageJ software (NIH).

### Endothelial cell apoptosis.

Small intestine endothelial apoptosis was examined by double staining with TUNEL for apoptosis and endothelial cell surface marker MECA32, as previously described (6, 51). Briefly, 5 mm jejunal sections were sequentially stained with TUNEL and MECA-32. Apoptotic endothelial cells display a brown TUNEL^+^ nuclear signal surrounded by a dark blue plasma membrane signal indicative of MECA-32 staining. At minimum, 2,000 endothelial cells were evaluated per point.

### IF studies of DSB repair foci.

Paraffin-embedded tissue sections (3 µm) were melted on a heat block, deparaffinized by 3 × 10 minutes in xylene, 2 × 3 minutes in 100% ethanol, 2 × 3 minutes in 95% ethanol, 2 × 3 minutes in 70% ethanol, before being washed with distilled water and transferred to PBS. Antigen retrieval was performed using boiled Antigen Unmasking Solution, Citric Acid Based pH 6.0 (Vector Laboratories, H-3300) in a Decloaking Chamber (BioCare Medical) at 125°C for 5 minutes, cooled for 30 minutes at room temperature, washed with distilled water, and transferred to washing buffer containing 0.1% Triton X-100 (Sigma-Aldrich, T8787) in 1× PBS. Foci were then probed using antibodies against established radiation-stimulated proteins overnight at 4°C. Prolong diamond anti-fade mountant with DAPI (Thermo Fisher Scientific, P36962) were used to protect from photobleaching and quenching of fluorescence signal, respectively.

### Antibodies used for IF and IHC studies.

Primary antibodies used include: mouse monoclonal antibody anti-γH2AX-Ser139 (Millipore [clone JBW 301], 05-636, dilution 1:1,000), rabbit polyclonal anti-BRCA1 [Ser 1387] (Novus Biologicals, NB100-225SS, dilution 1:400), mouse monoclonal anti–DNA-PKcs (phospho-T2609) (Abcam, ab18356, dilution 1:100), mouse monoclonal anti-MDC1 (MilliporeSigma, 05-1572 [clone P2B11], dilution 1:100), and MECA-32 (DSHB, MECA-32-s, dilution 1:10). Secondary antibodies used include: F(ab’)2-goat anti–rabbit IgG (H+L) cross-adsorbed secondary antibody, Alexa Fluor 488 (Thermo Fisher Scientific, A-11070, dilution 1:400) or F(ab’)2-goat anti-mouse IgG (H+L) cross-adsorbed secondary antibody, Alexa Fluor 488 (Thermo Fisher Scientific A-11017, dilution 1:400).

### Statistics.

Statistical analyses of experimental data were performed using GraphPad Prism software (version 8) and R (R 4.3.2; https://www.r-project.org/). Significance of differences was derived from 2-sided Wilcoxon rank sum or signed-rank tests, except in Supplemental Figure 2B where the Wilcoxon test would be underpowered due to small sample size; therefore, 1-sided, 2-sample *t* tests were used. To adjust for multiple comparisons, the FDR method was implemented. The significance level was adjusted according to number of tests conducted for each experiment. For tumor growth studies, a multivariate logistic regression was also run to examine the effect of dose level and group.

### Study approval.

Mice were housed at the Research Animal Resource Center of Memorial Sloan-Kettering Cancer Center, a facility approved by the American Association for Accreditation of Laboratory Animal Care and maintained in accordance with the regulations and standards of the United States Department of Agriculture and the Department of Health and Human Services, NIH. Protocols for conducting animal experiments were approved by the Memorial Sloan-Kettering Cancer Center Research Animal Resource Center.

### Data availability.

All relevant data supporting the findings in this study are available within the article and its supplemental files. Values for data points are reported in the Supporting Data Values file.

## Supplementary Material

Supplemental data

Unedited blot and gel images

Supporting data values

## Version 1 05/22/2025

In-Press Preview

## Version 2 06/23/2025

Electronic publication

## Version 3 07/03/2025

In the section describing the use of antibodies, “anti-MECA-32” was replaced with “MECA-32”
