# Supplementary material for: Radiosensitizing the SUMO stress response intensifies single-dose radiotherapy tumor cure
Source: JCI Insight. 2025 May 22;10(12):e153601. doi: 10.1172/jci.insight.153601 (PMC12220964; doi:10.1172/jci.insight.153601)
Supplement: Supplemental data [file jciinsight-10-153601-s008.pdf]

Supplemental Materials for

Radiosensitizing the SUMO Stress Response Intensifies Single Dose Radiotherapy Tumor Cure

Jin Cheng, Liyang Zhao, Sahra Bodo, Prashanth K.B. Nagesh, Rajvir Singh, Adam O. Michel, Regina Feldman, Zhigang Zhang, Simon Powell, Zvi Fuks and Richard Kolesnick[$]

[$]Correspondence: E-mail: r-kolesnick@ski-mskcc.org

This PDF file includes:

- Supplemental Table 1
- Supplemental Figure 1
- Supplemental Figure 2
- Supplemental Figure 3

Supplemental Table 1

Mouse number	Strain	Gross Finding	Final Diagnosis on Skin and subcutis
1	B6;129	Received a fixed tissue	Normal tissues (no evidence of neoplastic cells).
2	B6;129	Received a fixed tissue	Normal tissues (no evidence of neoplastic cells).
3	B6;129	Received a fixed tissue	Normal tissues (no evidence of neoplastic cells).
4	B6;129	Received a fixed tissue	Normal tissues (no evidence of neoplastic cells).
5	B6;129	Received a fixed tissue	Normal tissues (no evidence of neoplastic cells).
6	B6;129	Received a fixed tissue	Normal tissues (no evidence of neoplastic cells).
7	B6;129	Received a fixed tissue	Normal tissues (no evidence of neoplastic cells).
8	B6;129	Received a fixed tissue	Normal tissues (no evidence of neoplastic cells).
9	B6;129	Received a fixed tissue	Normal tissues (no evidence of neoplastic cells).
10	B6;129	Received a fixed tissue	Normal tissues (no evidence of neoplastic cells).
11	B6;129	Received a fixed tissue	Mild panniculitis, neutrophilic and histiocytic, multifocal (no evidence of neoplastic cells).
12	B6;129	Received a fixed tissue	Mild dermatitis, neutrophilic and histiocytic, multifocal (no evidence of neoplastic cells).
13	B6;129	Received a fixed tissue	Mild panniculitis, neutrophilic and histiocytic, with intrahistiocytic hemosiderin pigment, multifocal (no evidence of neoplastic cells).
14	B6;129	Received a fixed tissue	Normal tissues (no evidence of neoplastic cells).
15	B6;129	Received a fixed tissue	Normal tissues (no evidence of neoplastic cells).
16	B6;129	Received a fixed tissue	Mild dermatitis, neutrophilic and histiocytic, multifocal (no evidence of neoplastic cells).
17	B6;129	Received a fixed tissue	Mild panniculitis, neutrophilic and histiocytic, with intrahistiocytic hemosiderin pigment, multifocal (no evidence of neoplastic cells).
18	B6;129	Received a fixed tissue	Mild dermatitis, neutrophilic and histiocytic, multifocal (no evidence of neoplastic cells).
19	B6;129	Received a fixed tissue	Normal tissues (no evidence of neoplastic cells).
20	B6;129	Received a fixed tissue	Normal tissues (no evidence of neoplastic cells).
21	B6;129	Received a fixed tissue	Normal tissues (no evidence of neoplastic cells).
22	B6;129	Received a fixed tissue	Normal tissues (no evidence of neoplastic cells).
23	B6;129	Received a fixed tissue	Normal tissues (no evidence of neoplastic cells).
24	B6;129	Received a fixed tissue	Normal tissues (no evidence of neoplastic cells).
25	B6;129	Received a fixed tissue	Normal tissues (no evidence of neoplastic cells).
26	B6;129	Received a fixed tissue	Normal tissues (no evidence of neoplastic cells).
27	B6;129	Received a fixed tissue	Normal tissues (no evidence of neoplastic cells).
28	B6;129	Received a fixed tissue	Normal tissues (no evidence of neoplastic cells).
29	B6;129	Received a fixed tissue	Normal tissues (no evidence of neoplastic cells).
30	B6;129	Received a fixed tissue	Normal tissues (no evidence of neoplastic cells).
31	B6;129	Received a fixed tissue	Normal tissues (no evidence of neoplastic cells).
32	B6;129	Received a fixed tissue	Normal tissues (no evidence of neoplastic cells).
33	B6;129	Received a fixed tissue	Normal tissues (no evidence of neoplastic cells).
34	B6;129	Received a fixed tissue	Normal tissues (no evidence of neoplastic cells).

Supplemental Table 1. Complete responses represent tumor cure.
The Laboratory of Comparative Pathology at MSKCC examined 2 sections of skin from the tumor implantation site of a cohort of 34 mice that showed complete responses to SDRT in Figure 1. No evidence of neoplasia was detected in any specimen.

Supplemental Figure 1

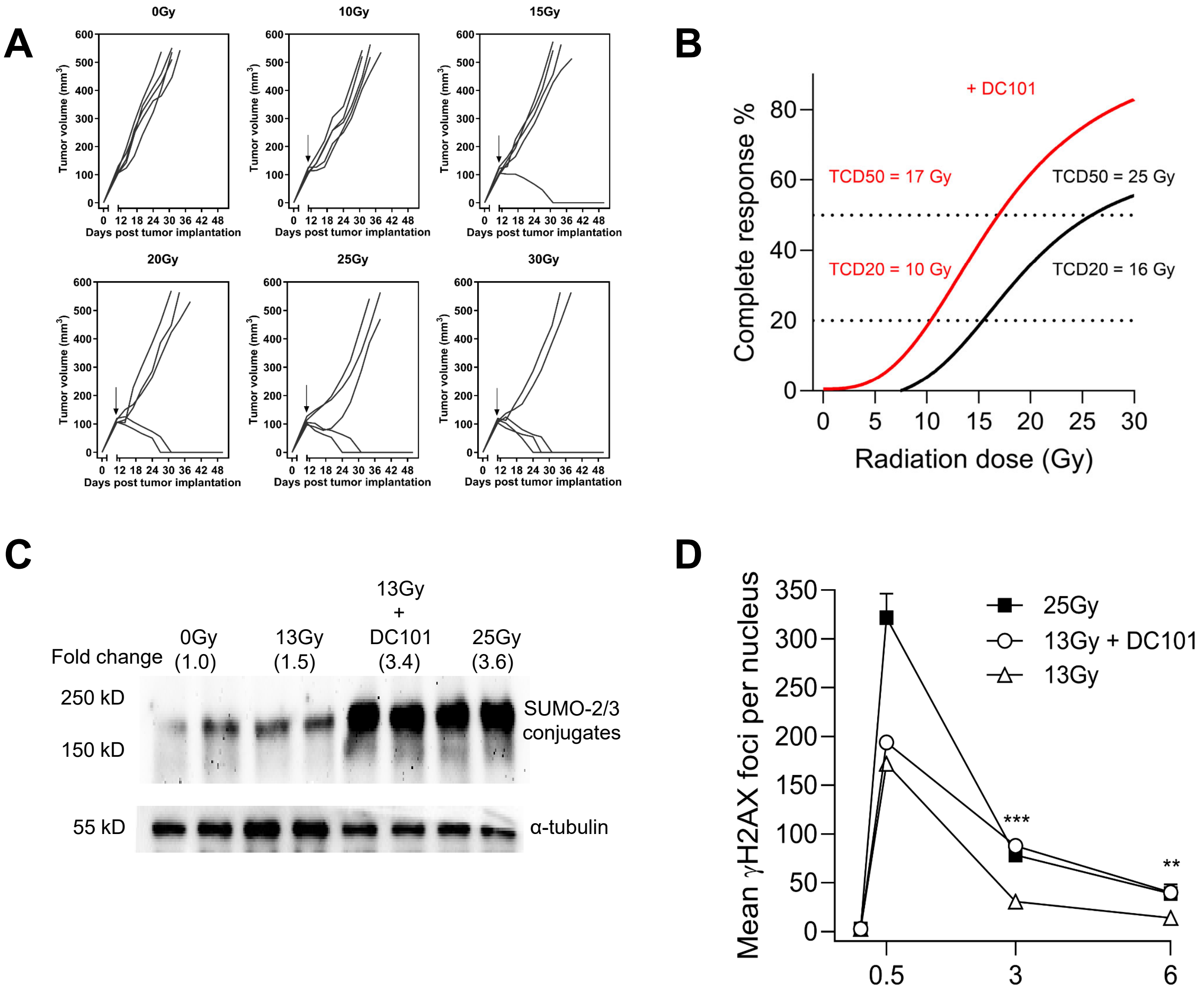

Supplemental Figure 1. Delivery of the VEGFR2 inhibitor DC101 to mice harboring Lewis Lung Carcinoma (LLC) xenografts at 1h preceding SDRT is radiosensitizing. (A) 1.5x10^6 LLC cells were implanted into the right flank of 8-10 wk old male sv129/BL/6J mice. Tumors (100-150 mm^3) were irradiated with SDRT at 10-30Gy. Tumor volume was measured 2x/week according to Kim et al. (Cancer Res 1986; 46:1120-3). Lines represent responses of individual tumors. Black arrows indicate day of irradiation. **(B)** Mice harboring LLC flank tumors were handled as in **(A)** with or without intravenous DC101 (1600 µg/25 gm mouse) pre-treatment at 1h preceding SDRT. Tumors were followed for 120 days and if undetectable at that time were considered complete responses. GraphPad Prism (version 10) was used for nonlinear regression analysis denoted by lines (sigmoidal curve fit) and quantified as tumor control dose (TCD) levels. DC101 yielded statistically significant sensitization of response (P=0.048, Wilcoxon signed-rack test on percent response from various dose groups with or without DC101). **(C)** Representative western blot image depicting high molecular weight (>100 kDa) SUMO2/3 conjugates (upper panel) contained in LLC flank tumor extracts isolated at 3h post SDRT. α-tubulin serves as loading control. **(D)** Accrual and resolution of γH2AX foci in LLC tumors with or without DC101 pre-treatment. Data represent mean±95%CI evaluating 3-5 high-powered fields (40x magnification) in 2 mice/group. **P=0.002, ***P=0.001 13Gy+DC101 vs.13Gy (2-sided Wilcoxon rank sum test).

Supplemental Figure 2

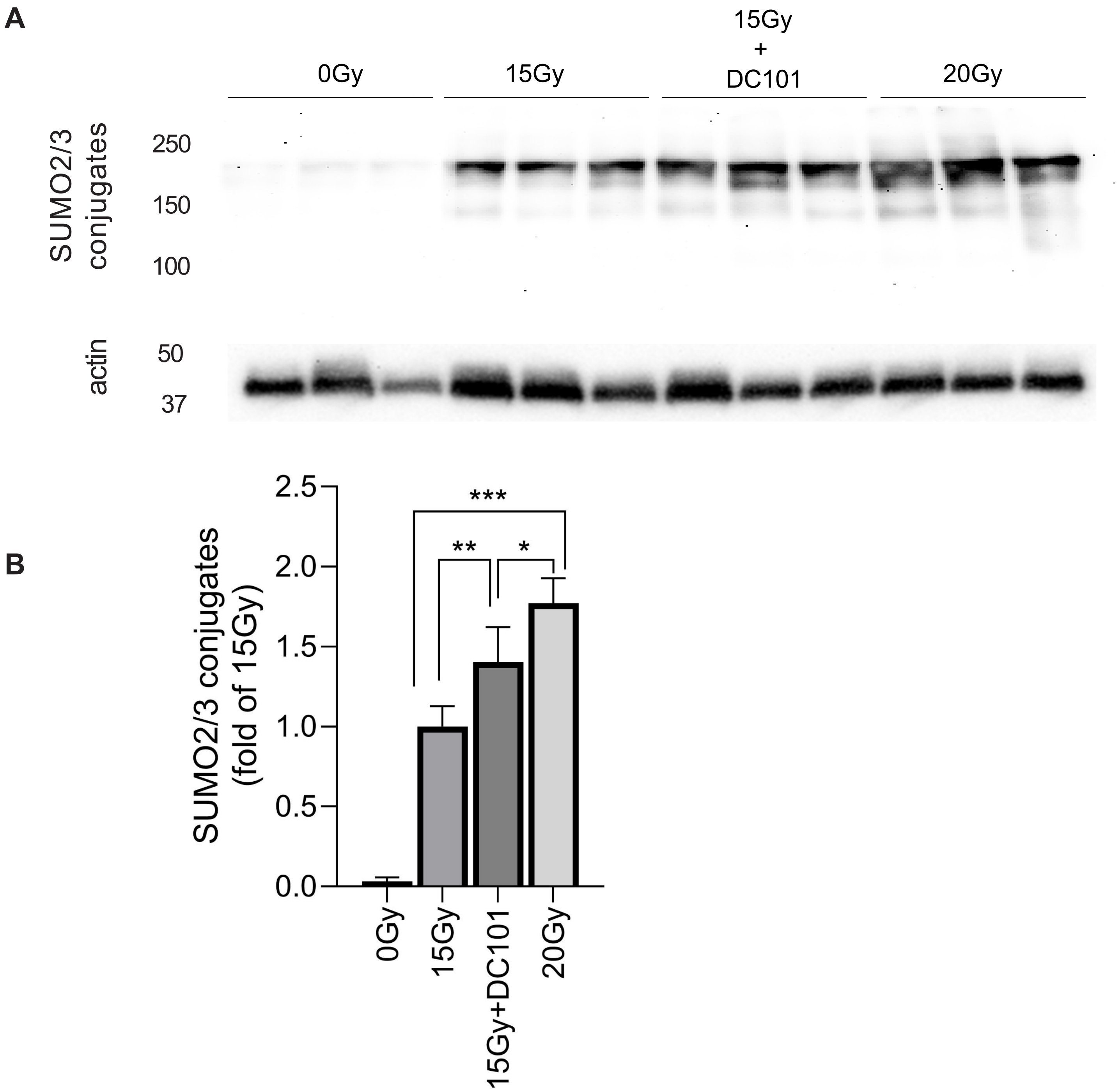

Supplemental Figure 2. Delivery of the VEGFR2 inhibitor DC101 at 1h preceding 15Gy SDRT sensitizes the SUMO Stress Response. (A) Representative western blot image depicting high molecular weight (>100 kDa) SUMO2/3 conjugates (upper panel) contained in extracts of MCA/129 flank fibrosarcomas isolated at 3h post 15Gy SDRT, with or without a 1h DC101 pre-treatment (1600 μg/25 gm mouse via tail vein injection), or 20Gy SDRT as in Fig. 1. **(B)** Quantitative analysis of high molecular weight (>100 kDa) SUMO2/3 conjugates. Data represent mean±SD using 3 mice/group each. *P=0.038, **P=0.025, ***P=0.001 (1-sided 2-sample t tests).

Supplemental Figure 3

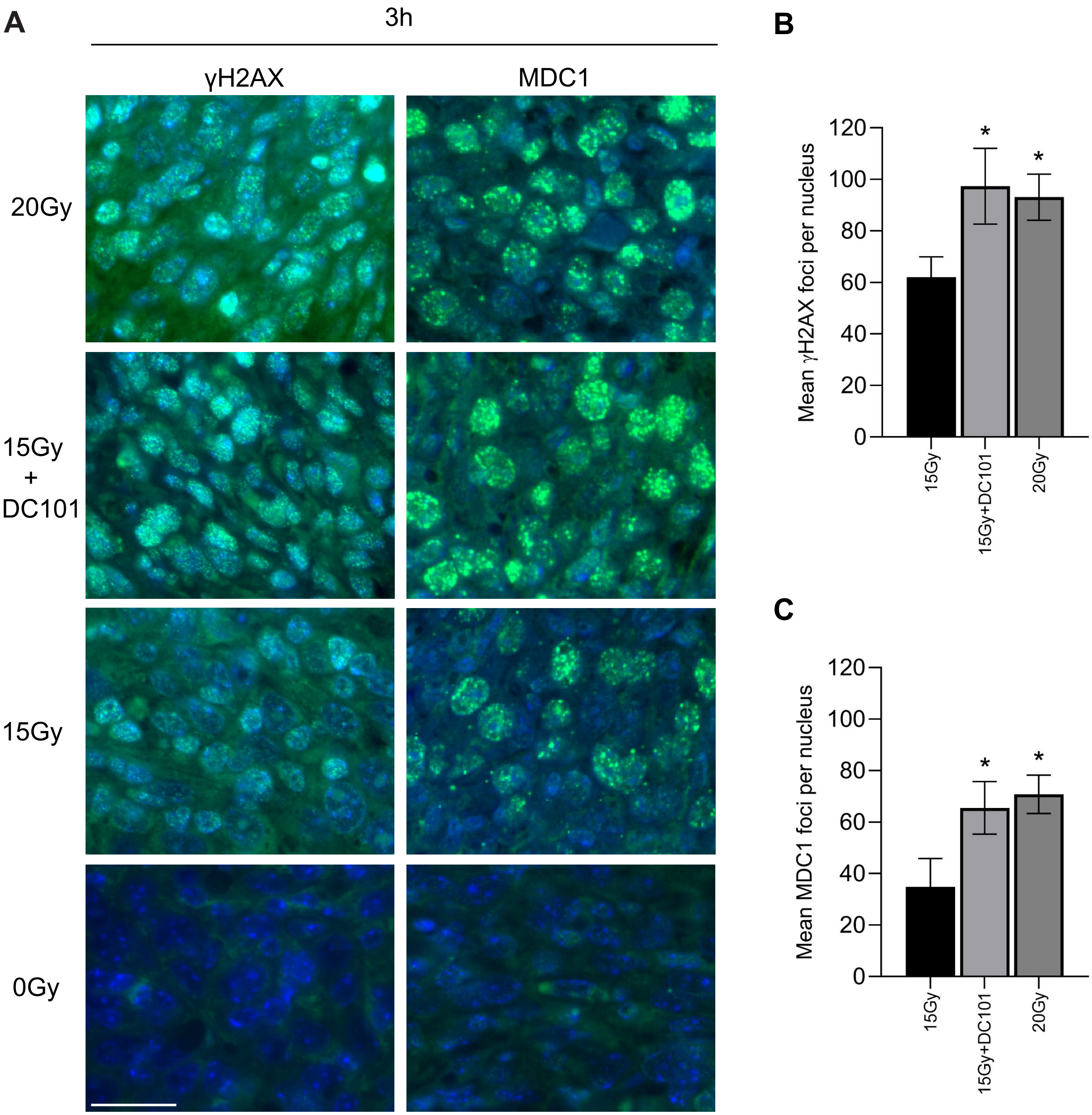

Supplemental Figure 3. Delivery of the VEGFR2 inhibitor DC101 at 1h preceding 15Gy SDRT attenuates γH2AX and MDC1 focus resolution at 3h post irradiation. (A) Representative γH2AX and MDC1 foci in MCA/129 flank fibrosarcomas treated with 15Gy SDRT with or without 1h DC101 pre-treatment or 20Gy SDRT as in Fig. 1. Scale bar: 20μm. **(B)** Data represent mean±95%CI, evaluating 3-5 high-powered fields (40x magnification) in 3 mice/group. *P<0.001 vs. 15Gy (2-sided Wilcoxon rank sum test). **(C)** Data represent mean±95%CI, evaluating 3-5 high-powered fields (40x magnification) in 3 mice/group. *P<0.001 vs. 15Gy (2-sided Wilcoxon rank sum test).
