# Supplementary material for: Radiosensitizing the SUMO stress response intensifies single-dose radiotherapy tumor cure
Source: JCI Insight. 2025 May 22;10(12):e153601. doi: 10.1172/jci.insight.153601 (PMC12220964; doi:10.1172/jci.insight.153601)
Supplement: Unedited blot and gel images [file jciinsight-10-153601-s009.pdf]

Full uncropped gels for Figure 2A

Mouse monoclonal
anti-SUMO2/3

Anti-mouse HRP

SSR

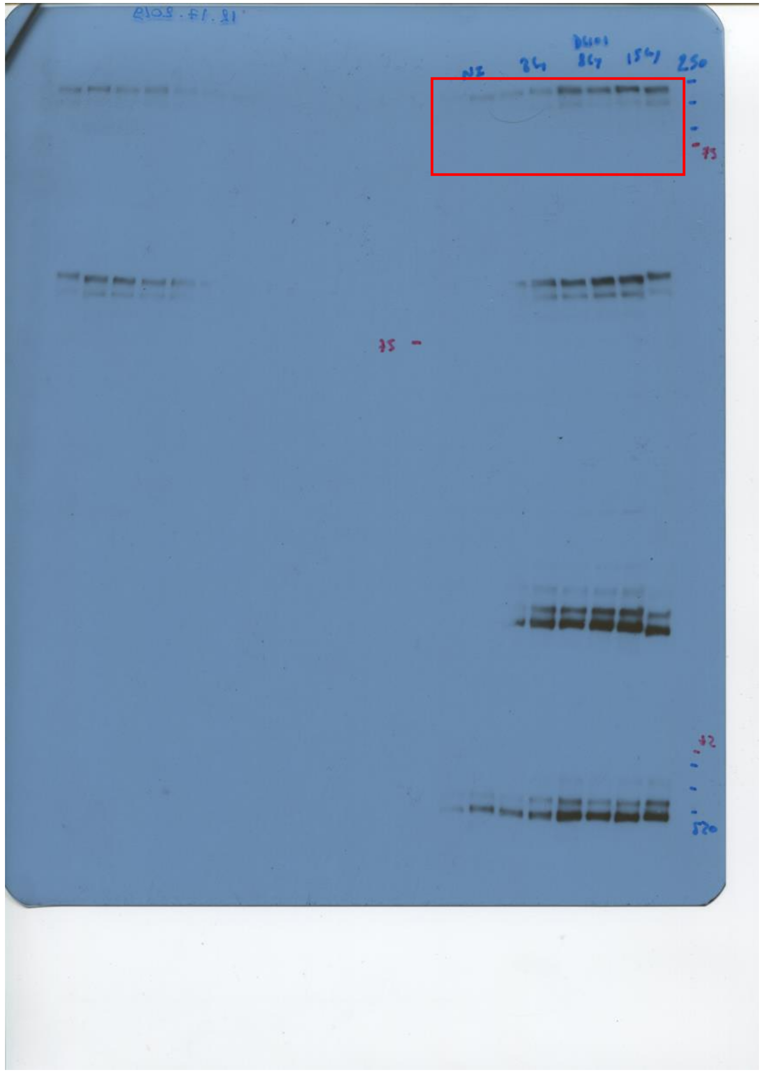

Actin

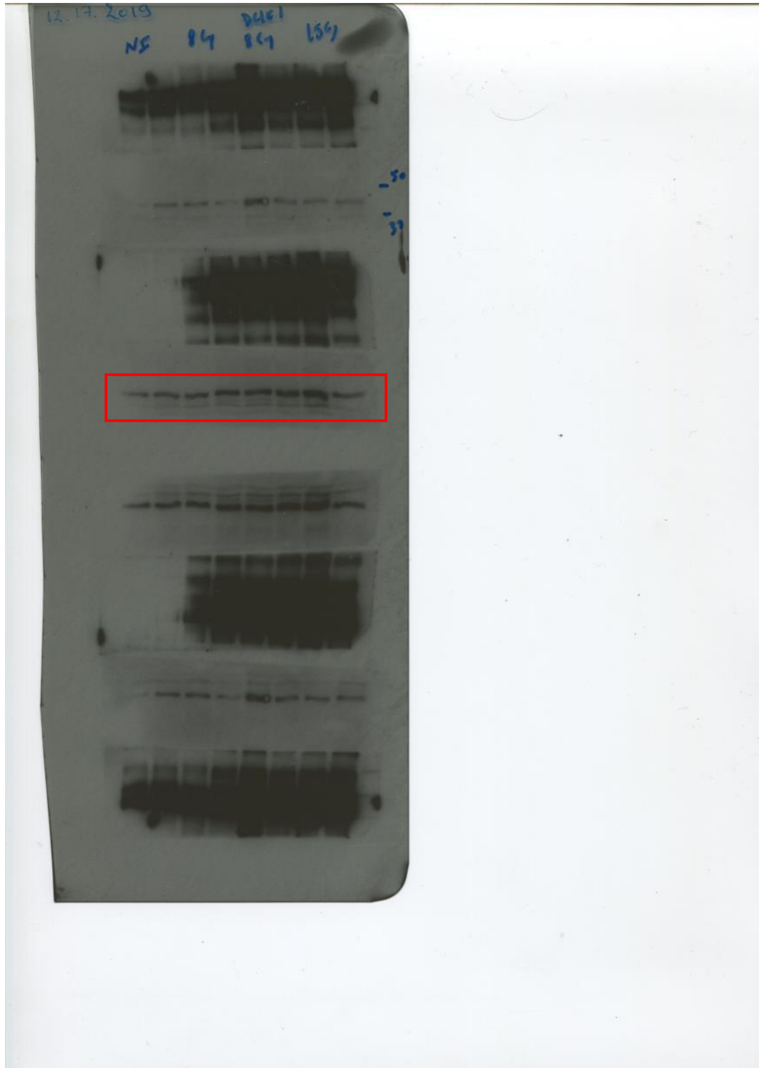

Rabbit polyclonal
anti-β-actin

anti-rabbit HRP

Full uncropped gels for supplemental Figure 1C

SSR

0Gy 13Gy 13Gy DC101 25Gy

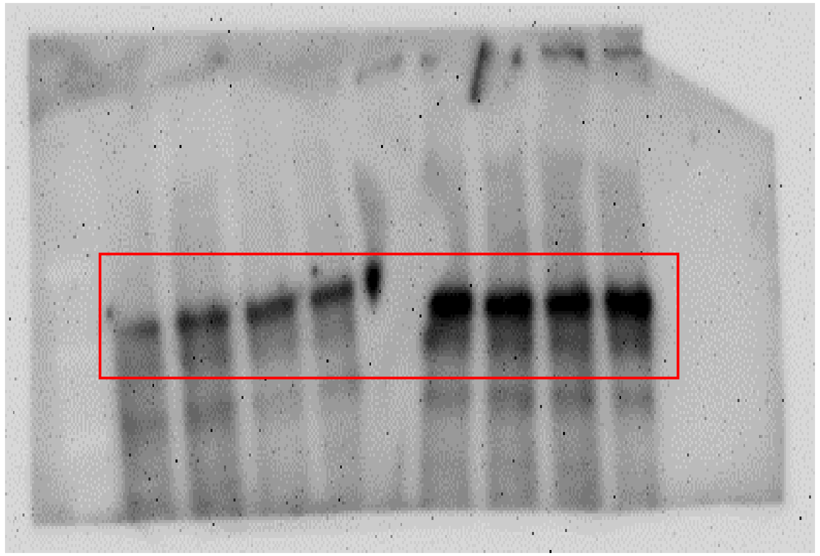

Mouse monoclonal anti-SUMO2/3

Anti-mouse HRP

α-tubulin

0Gy 13Gy 13Gy DC101 25Gy

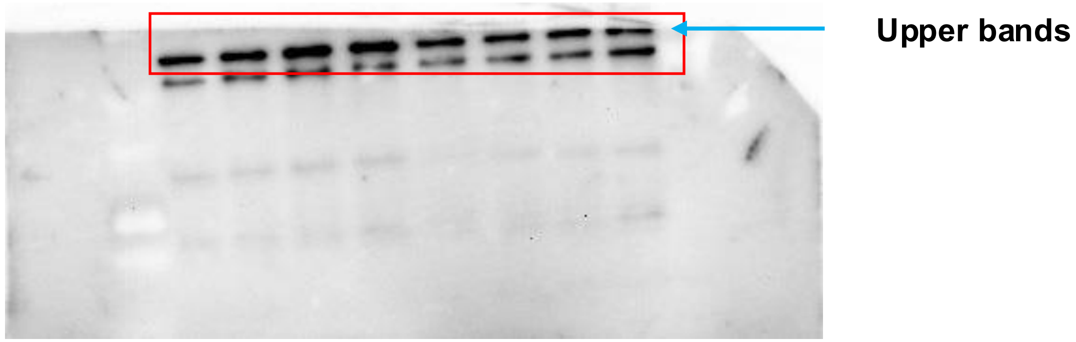

Mouse monoclonal anti-α-tubulin

Anti-mouse HRP

Full uncropped gels for supplemental Figure 2A

SSR

0Gy 15Gy 15Gy DC101 20Gy

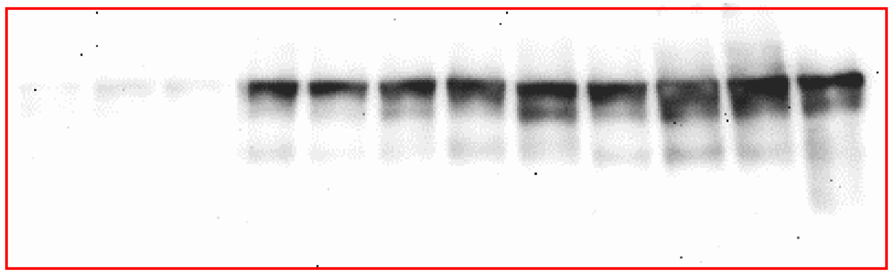

Mouse monoclonal
anti-SUMO2/3

Anti-mouse HRP

Actin

0Gy 15Gy 15Gy DC101 20Gy

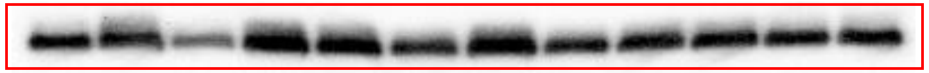

Rabbit polyclonal
anti-β-actin

anti-rabbit HRP
